# Youth Perspectives of Healthcare in Central Mexico: An Application of Massey’s Critical Health Literacy Framework

**DOI:** 10.3390/ijerph16050896

**Published:** 2019-03-12

**Authors:** Steven Hoffman, Heidi Adams Rueda, Lauren Beasley

**Affiliations:** 1 School of Social Work, Brigham Young University, Provo, UT 84660, USA; 2 Department of Social Work, University of Texas at San Antonio, San Antonio, TX 78207, USA; heidi.rueda@utsa.edu; 3 Knoxville Department of Kinesiology, Recreation, and Sport Studies, University of Tennessee, Knoxville, TN 37996, USA; lauren.beasley@utk.edu

**Keywords:** health literacy, adolescent health, public health, Mexico, health disparities

## Abstract

Attention to health literacy is essential more now than ever given the recognition, attention, and resources being dedicated to addressing health disparities throughout the world. Unfortunately, health literacy research is scarce in many parts of the world, particularly among youth. Using focus group discussions with junior high school students (*N* = 98) in a rural town of Central Mexico, we sought to learn about their experiences utilizing healthcare services at a local health clinic. The themes that naturally emerged from focus group discussions aligned with Massey’s framework on critical health literacy among US youth, and included problems navigating the health system, embarrassment speaking to doctors about sensitive issues, and minimal importance being placed on preventative care. This suggests that Massey’s framework may be appropriate to use when seeking to understand and promote health literacy among youth in Mexico. Furthermore, the challenges faced by adolescent participants in this study suggest that additional research is needed to assess how youth in other areas of Mexico are faring in efforts to understand and access their new and evolving universal healthcare system.

## 1. Introduction

Health literacy (HL) is an individual’s ability to gain, understand, process, and apply health information to make appropriate health decisions [1]. Health literacy has been found imperative for improved individual health. Many studies have shown a positive relationship between HL and health knowledge, such as knowledge of the negative effects of smoking and drinking and how to control chronic diseases such as HIV and hypertension [2]. Low HL, conversely, is associated with poor health, limited use of preventative care, and higher hospitalization rates [3]. Moreover, a recent literature review found that low HL is linked to low medication compliance and misunderstanding medical information, which helps explain findings linking low HL to decreased health among certain at-risk populations [4]. Unfortunately, the majority of HL measures, interventions, and research has been focused in the United States among primarily White populations [1,5,6]. Global HL research is essential more now than ever given the recognition, attention, and resources being dedicated to addressing health disparities throughout the world on an individual, community, and societal level [7,8,9]. A global effort to understanding HL is necessary when seeking to address the needs of migrant populations—particularly the growing migration networks connecting the U.S., Mexico, and Central America [8].

A focus on HL in Mexico is of particular importance at this time due to the country’s recent implementation of universal healthcare [10]. Through a series of nationwide health reforms and financial restructuring between 2003 and 2012, universal healthcare in Mexico, known as Seguro Popular, is now utilized by over 60 million Mexican citizens. Prior to the beginning of reforms in 2003, the Mexican healthcare system was decentralized, and it fell on specific states to provide healthcare to the uninsured population. Often this meant high out-of-packet expenses for low-quality care, while insured Mexican citizens received healthcare from well-funded federal hospitals and clinics. This created inequalities in health services between the insured and uninsured. The goals of the healthcare reforms were to offer the equivalent of the federal health services to the uninsured population, reduce out-of-pocket-expenses, and take some of the financial responsibilities off the states. There were a large number of changes under the healthcare reform (for a full overview of the policy and further statistics, see Knaul et al. [11]); the current system (Seguro Popular) now works as a public fund under the Ministry of Health that the government, the states, and individual citizens (based off income, with lower-income families exempt) all pay into. The fund covers essential healthcare services of enrolled citizens who do not have health insurance through employment, creating an option of health coverage for all Mexican citizens [11]. While citizens have the choice of whether or not to enroll, many states have elected to automatically enroll all their citizens as government funding for healthcare distributed to each state is based on enrollment numbers [11]. Since the implementation of Seguro Popular, use of health services by Mexican citizens of all ages has steadily increased [12], and the dramatic improvements in healthcare coverage and service delivery have been heralded by health watchdogs and governments throughout the world [13]. However, several limitations of healthcare access in Mexico still persist, especially in isolated rural areas where there are a lack of clinics, doctors, and relatively low patient–provider ratios [13,14,15]. Furthermore, vulnerable groups in Mexico such as women, the elderly, and low-income populations are still facing insurance gaps, and preventative care practices are not available for large portions of the population [16], as any services not deemed “essential” by the government are not covered under Seguro Popular [11].

Unfortunately, an improved health system does not necessarily lead to improved HL among consumers. In fact, a changing healthcare system could make navigating services more difficult for individuals [17], particularly those with low HL. For example, Gazmararian et al. [18] found that among those with access to universal healthcare in the U.S. (i.e., Medicare for the elderly), 23.5% of English-speakers and 34.2% of Spanish-speakers still had inadequate HL. Furthermore, citing statistics from a Kaiser Permanente survey, Levitt [19] discussed the implications of the Affordable Care Act (ACA) on newly enrolled Americans. Only 57% of the surveyed population understood the term “provider network” and only 53% could define “a deductible”, demonstrating the challenges facing new consumers trying to find their way within a complex health bureaucracy. Since individuals with limited HL inherently struggle to obtain care and make informed health decisions, assessing the HL of healthcare recipients should be a priority whenever there is a major shift in the structure of a national healthcare system. Unfortunately, at the time of this literature review, to our knowledge only one study has assessed HL in Mexico after the full implementation of Seguro Popular. Verastegui, De La Garza, and Allende-Perez [20] surveyed adults in an outpatient Mexican cancer clinic and found low HL in 15.4% of their sample, suggesting the vast majority had at least adequate HL. While their study provides important information for a unique subset of the population (adult patients with cancer), HL research is nonexistent among the majority of the Mexican population, including groups with historically low HL, such as youth [21].

An understanding of HL among youth is important because adolescence is a key development stage where children foster skills for adulthood [22]. Research suggests that low HL in youth—which can lead to the misinterpretation or misuse of health information—is correlated with adolescent obesity [23], less positive health habits [17], and an increase in risky behaviors, such as unsafe sex [24]. In developing nations, it is particularly important for youth to be able to discern between correct and erroneous health information as they typically have less access to the internet, fewer health professionals in their communities, and poorer health outcomes compared to youth in more developed regions of the world [25]. Although a few studies on adolescent HL have been conducted outside the US, the majority of HL studies among adolescents have focused on children in the U.S. [26].

One of the few studies that collected data outside of the U.S. was conducted by Hoffman and Marsiglia [25]. Using a sample of 230 adolescents, they explored the link between HL and substance use among youth in Guatemala. They found that those who went through a substance abuse prevention program scored higher on HL assessments than those who did not. Their results suggest that in more rural, developing parts of the world, utilizing existing health promotion programs and resources could be a viable, and economically beneficial, way to target and improve HL. Another global study offered a preliminary look at adolescent HL in Taiwan [27]. In her sample of over 1600 Taiwanese high school students, Chang found that adolescents with lower HL also had lower health status and poor health behaviors. Studies such as these are important foundational efforts, and similar efforts—within the context of critical HL—are needed in countries such as Mexico where information about the impact of healthcare changes is crucial for identifying systematic strengths and shortcomings in their new system.

In response to the growing recognition of the value of studying adolescent HL, frameworks with attention to adolescents specifically have been developed [6,28]. One such framework was developed by Massey, Prelip, Calimlim, Quiter, and Glik [29], who conducted 12 focus groups with 137 publicly insured teenage youth in the U.S., and completed 36 key informant interviews with primary care physicians. The authors’ goals were first, to clearly operationalize the growing definition of adolescent HL, which had lacked a clear and succinct definition in the literature [22], and second, to develop a framework that can be used to develop specific measures and scales of adolescent HL. They identified five prominent domains, each of which serves to operationalize an adolescent’s knowledge, attitudes, and practices in a healthcare setting: (1) their ability to appropriately navigate the health system (e.g., knowing how to make an appointment); (2) their knowledge and implementation of their own rights and responsibilities; (3) their knowledge of the need for and the implementation of preventative care practices; (4) their demonstration of appropriate and accurate health information seeking practices; and (5) their ability to form positive and effective patient–provider relationships. Competence in each of these domains can help to define the extent to which an adolescent is “health-literate”, as utilizing an operationalization of adolescent HL with multiple domains can better capture the varying definitions of HL [29]. With this expanded framework, policy makers and stakeholders may be able to better identify where gaps in services exist.

This model in particular serves as a useful lens for analyzing adolescent HL as it focuses on many aspects of critical HL, specifically on how adolescents actually interact with health systems. This is becoming more imperative as more and more adolescents gain access with the expansion of universal healthcare worldwide [29]. Critical HL differs from other aspects of HL (e.g., document, quantitative, and functional HL) in that it centers on the ability of an individual to apply their health knowledge in a manner that allows them to exert control over their health [30,31,32]. Chinn [31] defines three unique aspects to critical HL: information analysis and appraisal, social/structural aspects of health, and collective action. While HL addresses an individual’s ability to obtain health knowledge through reading, listening, or searching out information, critical HL is application and action-focused, and thus is a more robust framework for assessing the HL of populations or groups as it points to the ability to actually use information to improve one’s health [33]. Within Massey et al.’s [29] model for adolescent HL, there is an underlying focus on actions adolescents can take to gain control over their health; specifically, asking questions of their doctors, taking appropriate steps to access their healthcare, and making preventative health decisions, which may ultimately improve their health. This focus on action also incorporates the growing efficacy and control adolescents have over their healthcare [22]. Massey et al.’s model [29] may also serve as a guide for cross-cultural research because it focuses on actual health behaviors, which allows for more attention to variation across cultures, versus solely health knowledge of adolescents, which many times is measured through comprehension only [22] and, subsequently, with stakeholders in policy considerations aimed narrowly at increasing adolescent knowledge. Considering the changing landscape of healthcare in Mexico, use of Massey et al.’s framework, which specifically focuses on interaction with the health system, may be able to provide valuable insights concerning the assessment, promotion, and actions taken by adolescents with regard to HL. Therefore, the purpose of this study was to assess the fit of Massey et al.’s framework of critical HL among Mexican youth by exploring their experiences utilizing healthcare services at a community health center. In doing so, we recognize the inherent limitations of applying a US derived model to Mexican youth, namely that the US and Mexico have very different health systems and distinctive norms and expectations surrounding healthcare utilization. Rather than comparing findings across cultures, we focus on the extent to which Massey et al.’s model fits, which may provide a useful framework for understanding Mexican youth experiences in their own right. Expanding our understanding of adolescent HL and health-seeking behaviors in Mexico is a foundational step towards understanding the potential impact of a changing healthcare system on their ability to meet their personal health goals.

## 2. Materials and Methods

### 2.1. Study Context

Procedures for this study were approved by the governing Institutional Review Board (UTSA Approval Number: 14-241N). We worked closely with two school psychologists from a middle school in a rural town of Central Mexico to recruit youth to participate in a two-pronged study involving a short survey and a focus group about health. Prior to conducting the study, we trained the school psychologists remotely using Skype in all data collection procedures. All youth in the school were eligible for recruitment and were recruited by the psychologists. As migration (both legal and illegal) to the US is integral to the financial stability of the community, the disclosure of personal information to a binational research team was concerning to members of the community. Thus, the use of active and written consent was determined to be unethical and unnecessary by the research team. Alternatively, the school psychologists spoke with parents and youth to answer questions about the risks and benefits of study participation and to discuss confidentiality. Specifically, parents and youth were told that researchers from a university in the U.S. were interested in youth’s health and health-related experiences. After discussing the study, any parents or youth could opt to not participate further.

### 2.2. Sample

Of the 116 potential student participants, four chose not to participate in either the demographic survey or the focus groups. These students dropped out of school during the recruitment process, which was reported as not uncommon among students in the area. As shown in Table 1, 112 students completed the demographic survey. Of the 112 students, 98 also participated in a focus group (61 males and 37 females).

### 2.3. Procedures

Eight focus group (five male and three female) took place during normal school hours. The school psychologists worked with teachers to remove students by grade and sex from classrooms to attend the focus groups. One psychologist was male and the other female and each moderated their respective same-gender focus groups. Each discussion lasted approximately 30–45 min. Focus groups were large, averaging just over 12 students. Youth were asked a variety of health-related questions, including where they received health information, their experiences at a local health clinic, and who they spoke to about their health (see Appendix A for a full list of discussion questions).

### 2.4. Data Analysis

Data from the focus groups were audio recorded, which were later translated from Spanish to English and transcribed by a bilingual member of the U.S. research team. Translations were then checked by a second bilingual member of the research team, an independent bilingual reviewer (not part of the research team), and a bilingual member of the data collection team in Mexico. The English language transcripts were entered into NVIVO, a qualitative software program (QRS International, Melbourne, Australia) [34]. We followed the steps outlined by Braun and Clark [35] in first familiarizing ourselves with the data, open coding the transcripts inductively to note salient and reoccurring patterns, and categorizing these patterns into collapsed and meaningful themes via a preliminary codebook. That is, the codebook was developed in order to operationalize themes which had arisen from the data in a manner of frequency, specificity of discussion (e.g., examples and elaboration), and emotionality. This was in alignment with Krueger and Casey’s [36] best practice recommendations for analysis of focus group data. As is common in qualitative research (see Crabtree & Miller [37]; Padgett [38]), we were sensitized through our preliminary coding process to the overlap between initial themes and those outlined by Massey et al.’s [29] framework for studying adolescent HL (e.g., our theme pertaining to “barriers to positive relationships with doctors” with theirs of “patient–provider relationships”), and thus moved from an inductive to a deductive codebook in order to assess fit of our data with this useful framework and to outline findings in a salient context to healthcare literature. (For an example of this inductive to deductive template approach, see Linton & Rueda [39].) The fit of our preliminary codebook to Massey et al.’s framework was excellent to the extent that only the titles and order of themes were changed. An inter-rater reliability kappa was calculated on the final codebook with a fourth and independent researcher who coded the data in its entirety. We had excellent reliability between the initial coding and that of the independent researcher who was unfamiliar with the study or its aims (*κ* = 0.93). Throughout the analysis process, the rigor of the study was further enhanced by use of observer triangulation and member checking. That is, three separate researchers corroborated to analyze the data [38] and the final themes of the study were sent to the school psychologists in Mexico for their feedback. Their feedback resulted in some minor changes to wording within the results section to capture accurate translations stemming from local colloquialisms. We report the following themes from our data in alignment with Massey’s [29] framework of adolescent HL: (1) navigating the health system, (2) patients’ rights and responsibilities, (3) preventative care, (4) information seeking, and (5) patient–provider relationships.

## 3. Results

The purpose of our study was to explore the experiences of youth in a rural Central Mexican town concerning their health experiences including those at a local healthcare clinic. Here, we outline themes in accordance with Massey and colleagues’ [29] framework for critical health literacy. Direct Spanish quotations are provided alongside examples. Students chose their own pseudonyms at the start of each focus group.

### 3.1. Navigating the Health System

Although students knew the basics of how their local health clinic worked, they expressed frustration in not being able to get appointments or appropriate medications due to systematic issues with the center. The center utilized a token system that determined the order in which patients were seen by the doctors. Many students felt that the token system was ineffective, particularly as it disallowed them from seeing a doctor despite the fact that they had visited the clinic. Roxy (age 15, female) gave a response indicative of the frustration of many other students, stating “…at eight in the morning they give tokens. If you make it, good! If not, oh well (O que a las ocho de la mañana dan las fichas, si alcanzaste bien si no, ni modo).” Gordito (age 14, male) explained that they do not give out many tokens a day, “they give out very few tokens, thirteen or ten (Dan muy poquitas fichas, trece o diez).” As Natalia (age 12, female) explained, if you do not get one of those ten tokens you have to “come (again) tomorrow (Venga hasta mañana).” The frustration with the token system was exasperated by the limited hours of the health center. The hours of the pharmacy were also limited, closing at noon, and making it difficult for students to get their medicine without missing school or their caretakers missing work. Halo (age 13, male) elaborated, “or sometimes they have closed, but you ring the bell and they do not open (O hay veces que tienen cerrado y tocas el timbre y no te abren).” Furthermore, even if they were able to see a doctor and make it to the pharmacy, many students expressed that the center did not have the medication they needed available: “there are almost no medications (Que no hay casi medicamentos)” (Isabela, age 14, female).

### 3.2. Patients’ Rights and Responsibilities

Patient responsibility was brought up by the youth in the context of asking clarifying questions of the doctor if information was not readily understood. Students expressed that many times they did not understand the medical language doctors used: “they talk about things we do not understand (O que hablan de cosas que nosotros no entendemos)” (Pancracio, age 15, male); “it’s that you do not understand how they explain things (Es que no entiendes como te explica por sus palabras)”(Bart, age 13, male). Estrella (age 14, female) said that patients have to ask the doctors to “can you please tell us more clearly what we have, or whatever?’ (Nos puede decir por favor más claro lo que tenemos o ¿equis coas?).” Axel (age 13, male) stated that when one goes to the doctor the patient has to “ask them [the doctor] what sickness you have and to please explain it. Why did I get sick? (Tú le vas decir qué enfermedad es esa y me la puede explicar por favor y porque esa enfermedad me dio).” The youth clearly understood that, as patients, they have the right to be spoken to at a level and in a way that they can understand. However, as will be discussed further in the theme of patient–provider relationships, barriers to communication with health professionals included fear, mistrust, and embarrassment, which seemed to stem mostly from discussion of sexual health. It may be that the youth would feel more comfortable asking these questions of their doctor if not related to their sexual health. Even so, although many students spoke about barriers to communication with their doctor, some of the same students still spoke to the importance of asking these clarifying questions.

### 3.3. Preventative Care

In response to being asked when they go to the doctor the participants’ answers overwhelmingly supported a nonpreventative approach to healthcare, with many saying they only go to the doctor “as soon as you’re feeling pain from the sickness (En cuánto te da el dolor de la enfermedad)” (Axel, age 13, male); “well, only when we have the flu (Pues solo cuando tenemos gripa)” (Aureli, age 16, male); and “when you feel bad (Cuando te sientes mal)” (Lena, age 14, female; Shakira, age 16, female). A few students demonstrated a somewhat more preventative mindset, stating that one should go to the doctor either “when you have some doubt (Cuando tienes alguna duda)” (Ron, age 13, male) or “when you start to show symptoms (Cuando empiezas a presenter síntomas)” (Panfilo, age 15, male). Only one student (Teo, age 13, male) stated that one should go to the doctor “every month, every two months, or at least every year (Cada mex, cada dos meses o máximo cada año).”

### 3.4. Information Seeking

Although the participants overwhelmingly indicated that they would go to the health center for health information, when asked who they talk to about their medical concerns only a few of the youth said they would seek out their doctor. The youth responded that they mainly go to their mom or extended family, including grandparents, aunts, uncles, and/or cousins. Other responses included their dad, siblings, teachers, or the internet.

### 3.5. Patient–Provider Relationship

Many participants described the health center’s medical staff as rude and not attending to patients, specifically pointing to the perceived lack of professionalism of the staff. Bart (age 13, male) offered his own experience as an example: “…when a (female) doctor is there and you say, ‘oh my, that doctor is going to be there’, and you go and they see you and say, ‘I’m busy’ and she isn’t busy and is actually on her phone and like, what? She’s not busy? (Cuando está una doctora y dices ‘chin, va a estar esa doctora’, y vas y te atiende y dice ‘estoy ocupada’ y en realidad está en el celular y ah, ¿cómo? ¿No está ocupada?)”. Gordito (age 14, male) sarcastically expressed his perspective of the doctor’s attitudes: “Let’s see if the doctor is not tired and wants to see you (A ver si el doctor no esta cansado y te quiere atender).” Rosita (female, age 13) mirrored this sentiment, expressing that she believed doctors at the health center “sometimes they do it with a bad attitude (A veces hasta con mala gana).” Aside from not attending to the patients, some students suggested the doctors were incompetent because they would not prescribe the correct medication, or because they would give shots incorrectly or in a way that hurt patients. Regardless of the accuracy of such statements, it is important to note how the doctors’ professionalism and competence were perceived by youth in the community.

A follow-up question seeking additional information about the challenges youth faced while speaking with the doctors led to a broad range of response including being scared of the doctor, not trusting the doctor, and simply not wanting to talk to the doctor. Several youth directly stated they were afraid of the doctor: “It scares me (me da miedo)” (Mayte, age 12, female); “I am afraid of the doctors (A mi me dan miedo los doctores)” (Victoria, age 12, female). Others stated that they do not talk openly with the doctors “because I do not trust them (Porque no les tengo confianza)” (Komander, age 13). The distrust theme was especially pervasive among female students, who were uncomfortable with male doctors. The female moderator summarized a conversation among the girls in one focus group: “Ah, yeah. There is no trust because they are doctors and in the case of the women [female youth], they are always hoping for a female doctor (Ah, ya. Que no hay confianza porque son doctores y en el caso de las mueres siempres buscan como una doctora).” Kalel (age 14, male) brought up how being scared and uncomfortable at the health center affected his communication with the doctor, stating, “we are really nervous, so we do not understand much (Estamos tan nerviosos que no entendemos mucho).”

When asked about their level of comfort speaking with adults, including doctors, about their health, the most common response was that it was difficult because they were embarrassed: “I am embarrassed with doctors (Me da verguenza con los doctores)” (Natalia, age 12, female); “…we get embarrassed (Que nos da pena)” (Lena, age 14, female); “we even get embarrassed to answer [their questions] (Hasta nos da verguenza contester)” (Gordito, age 14, male). For both males and females, the embarrassment many times stemmed from discussions of sexual health. Axel (age 13, male) explained “for example, sometimes your intimate [private] part hurts and you do not want to tell the doctor so they don’t check it (Por ejemplo. A veces te duele tu parte íntima y no le quiere decir al doctor para que no te la revise),” while Victoria (age 12, female) suggested that she was embarrassed because “they ask you…if you have had sexual relations (Igual te pregunta si has tenido relaciones sexuales).” Both male and female students expressed that having a doctor of the opposite sex contributed to their embarrassment. Aureli (age 16, male) commented “if a female doctor is chosen you get nervous (luego si te toca un…una doctora, te dan como nervios),” while Natalia (age 12, female) recounted her experience with a male doctor, “you feel a lot of embarrassment because, um, they tell you ‘raise your blouse a little bit so I can check your stomach,’ and you say like…ay…thinking ‘oh, no, how embarrassing’ (sientes como mucha pena porque, este, te dice: ‘Alzate la blusa tantito para checar tu estómago’, y te quedas como…ay…pensando ‘ay, no, que verguenza.’”

## 4. Discussion

The stories and experiences of youth seeking health information and care are integral to appraising their critical HL skills and health-seeking behaviors. This type of research is imperative to discussions on reducing health disparities, particularly in rural towns where health outcomes are often worse than urban or suburban areas due to the lower socioeconomic status of residents, poor service availability, and more hazardous living conditions [40]. Although research has suggested that Mexicans from low socioeconomic backgrounds have benefited in some ways from the universal healthcare system (i.e., Seguro Popular [12]), the present study offers a qualitative exploration of youth perspectives in the context of a rural Mexican town and details the lived experiences of struggle with the local health clinic provided under the Seguro Popular healthcare model. Theoretically, these results offer support for using Massey et al.’s [29] framework for analysis of adolescent critical HL in Mexico. On the one hand, the youth indicated they were able to navigate the system and understood the basic rights of the patient to be understand and applied these rights by asking clarifying questions of their doctors. However, they also spoke to a lack of basic preventative care practices, information seeking outside of the family, barriers to a productive relationship with healthcare professionals, and preferred family and other nonformal sources when they sought health information. An understanding of the strengths and limitations of the youth’s critical HL can guide interventions to increase competence in each of the framework’s five domains.

Participants in this study mentioned several barriers to developing a positive relationship with doctors including embarrassment, fear, mistrust, and doctor’s use of medical language. These are barriers to critical health literacy as outlined by Massey et al.’s [29] model and common concerns for adolescents across cultures. Two recent qualitative studies in the US found that the use of non-age appropriate medical language was one of the biggest barriers in effective communication between adolescent cancer patients and their doctor [41,42]. Embarrassment is also a common barrier to healthcare utilization by adolescents [43], and one of the main reasons youth turn to the internet to research health problems rather than making an appointment with their doctor [44]. As the youth in the present study suggest, sexual health specifically seems to lead to feelings of embarrassment and hesitation to speak to a healthcare professional [45]. Feedback from member checking with the Mexican psychologists reiterated sexual topics as particularly difficult for adolescents to broach with healthcare providers and their families, despite the developmental importance of these to youth. While fear and mistrust do tend to impact the patient-doctor relationship and healthcare utilization among youth across cultures [45,46,47], these two themes are particularly prevalent within samples of Hispanic adolescents [47,48]. For example, in a sample of immigrant Latino adolescents in the U.S., qualitative results suggested that mistrust of doctors stemmed from fear of deportation, cultural misunderstandings, and negative experiences with other authority figures, such as teachers [49].

Although youth in the sample overall understood how to utilize the health clinic in their community, there was less understanding of how to navigate the barriers inherent in the system, which is a key element of an adolescent competent in the service navigation domain of Massey et al.’s [29] model. Even though their understanding of how to navigate the barriers inherent in the healthcare system may be somewhat limited, their ability to identify system limitations is a strength that should not be overlooked. This skill is certainly more aligned with critical HL than functional HL, although it is not directly accounted for in Massey et al.’s model [29]. The ability to identify limitations coupled with the apparent inability to navigate barriers suggests that adolescence may be the ideal time to promote critical HL skills. While it may be tempting to think that functional HL skills should be developed at a young age followed by the development of critical HL skills in early adulthood, it may be that critical HL skills would be most beneficial if taught simultaneously alongside functional HL at a young age.

The systematic barriers of the healthcare clinic—specifically the poor customer service, unprofessionalism of clinical staff, and the token system—mirror many of the same issues that were targeted by the Mexican Ministry of Health prior to implementation of Seguro Popular [50]. In a 2001 report based on samples of Mexican healthcare consumers, the Mexican Ministry of Health touted low emergency room wait times, high satisfaction with care, high levels of staff kindness, and positive experiences scheduling appointments. However, only 25% of the hospitals and clinics surveyed were public entities, suggesting that the findings were, in essence, a report on the private healthcare sector. The public health system, which has dramatically grown since the 2001 report due to the implementation of Seguro Popular, is an altogether different entity, and the experiences of youth in this study seem to suggest that healthcare utilization issues may be of concern in rural Mexico.

Preventative care practices that involved seeing a doctor were very rare among youth in our sample. In fact, only one person mentioned going to the doctor when they were not sick. Low use of preventive care practices in Mexico is not unique to youth in this study. An Organisation for Economic Cooperation and Development [13] report cites the lack of preventative care as a major issue facing the current Mexican healthcare system, especially in rural parts of Mexico. One study, analyzing data from a public Mexican health survey, found that over 30% of patients requiring inpatient medical services did not have access to their needed health service [15]. As rural sates in Mexico have some of the lowest patient–provider, patient–nurse, and patient–paraprofessional ratios in the country [14], there is limited access to primary care providers in these rural towns, which makes preventative care visits difficult [13]. Decreasing barriers to access and increasing knowledge of the importance of preventative care practices is key to increasing adolescent HL [29].

Aside from issues around access, expectancy theory can also shed light on how the barriers to positive patient–provider relationships as the systemic issues present at the clinic may relate to a lack of preventative care practices by adolescents. Vroom’s [51] expectancy theory postulates that the motivations driving behaviors are linked to expected values and outcomes. Academic healthcare literature commonly uses expectancy theory to interpret patient expectations, perceived service quality, and satisfaction in both adult and adolescent populations [52,53,54,55,56]. Comments throughout our focus groups suggested that the local healthcare clinic has a bad reputation, and youth have had various negative interactions with the doctors and nurses. Expectancy theory suggests that this may lead to a diminished motivation to utilize the local healthcare clinic, with youth perhaps more likely to turn to less reliable sources for information with their healthcare questions and concerns (i.e., family members and the internet).

There are clear systemic changes that could improve the experiences of patients at the health clinic and increase service navigation and utilization. Changes to the token system (e.g., implementing an appointment-based system) could decrease long wait times. Changes to the operating hours of the clinic would better meet the schedules of adolescent patients in school (i.e., extending pharmacy hours beyond 12:00 pm). Frenk, Gomez-Dantes, and Knaul [57] identified long wait times at both outpatient clinics and hospitals, and lack of care hours during evenings and weekends as some of the major challenges facing the Mexican healthcare system after the implementation of Seguro Popular. The qualitative results of this study support the need for these areas, within the service navigation domain of Massey et al.’s [29] model, to continue to be explored by healthcare professionals and lawmakers in Mexico, particularly among rural healthcare providers.

On a community level, there are a variety of changes that could reduce the negative expectations of adolescents towards the local healthcare clinic, increase critical HL, and improve the utilization of health services. According to the short demographic survey completed by study participants, 64% of the students had attended a health class. To target the population who had not yet attended a health class, policy changes could require health education classes for all students in primary school, similar to what many states have already done in the U.S. [58]. Within the health class curriculum, a specific section or focus on patients’ rights could also increase competence in the rights and responsibility domain of adolescent HL [29]. Furthermore, to decrease negative views of doctors, local physicians from the health center could be featured as guest speakers during the required health classes. This would give them an opportunity to provide an overview of the medical services offered through the local clinic and answer student questions. Various studies suggest that increased familiarity with doctors can enhance the patient–provider relationship and provide more positive healthcare experiences for youth [59,60,61]. Within the context of Massey et al.’s [29] model, interventions to increase positive patient–providers relationships are key in increasing adolescent HL.

Finally, a large number of the youth stated that they turn to a parent or family member for health information. Although this certainly speaks to the importance of immediate and extended family in the Mexican culture [62], it is imperative to consider the quality of the advice they are receiving, especially as appropriate information seeking and the ability to critically examine health information is an essential skill of adolescent HL [29]. Due to the increased availability of the internet, it is critical that youth and parents know about legitimate online resources and websites that can provide accurate information [63]. Furthermore, much like other school subjects such as math [64], if health information from presentations at school are not reinforced at home, it is unlikely to be retained by youth. Incorporating parents into the health education of adolescents is considered a promising model [65], so parents should be offered resources from school or provided the opportunity to attend health classes similar to what their children are receiving. This would reinforce student learning, help parents become more health literate, and increase both students’ and parents’ ability to critically analyze the health information they are receiving [29]. Further, incorporating family and the community was deemed important in feedback from our Mexican research psychologists who communicated that school is not valued by many students who often did not attend class or who dropped out altogether.

Returning to the Massey’s [29] model for adolescent HL, our results suggest that this could act as a cross-cultural framework to explore the critical HL/health behaviors of adolescents, as the original thematic analysis fit within Massey’s framework. There were, however, clear cultural differences of themes within each dominion between our focus groups in Mexico and the American context of Massey et al.’s model [29]. For example, while the youth in this study spoke about issues with the token system, adolescents in Massey’s interviews spoke to the difficulty in getting an appointment time, which show the differences between the Mexican and American healthcare system. Perhaps the biggest cultural difference was the inclusion of parents, and especially extended family, in healthcare. While the American youth in Massey’s interviews viewed parents as gatekeepers to their healthcare and did not bring up the role of extended family [29], the Mexican youth spoke specifically to the role of the extended family in their healthcare. Therefore, the model could serve by including a domain on the role of social networks (including peers, family, extended family, and extended kin), especially in international research on adolescent HL, to gain a fuller understanding of the role family has in adolescent healthcare. Overall, though the domains fit well with the themes that emerged, they also provided a framework to organize the aspects of critical HL of adolescents in rural Mexico, which can guide future policies and interventions.

## 5. Conclusions

A qualitative methodological approach has inherent limitations, including the limited transferability of findings and inability to provide statistical support for policy and practice recommendations. Furthermore, this particular study had a relatively small sample size and rather large focus groups. Although smaller groups may have afforded more opportunity for individual discussion and cohesion, Toner [66] discusses the importance of conducting groups of various sizes with understudied populations. In doing so, this project provides key insights about the critical HL skills of an understudied sample of youth in Central Mexico, and expands on and supports a current framework for studying adolescent HL [29]. Moreover, this study offers a unique understanding into the negative perceptions of a local healthcare clinic among youth—perceptions that are influential in shaping their future utilization of proper medical services and trust in health professionals. Whether these negative perceptions and experiences are common among youth throughout Mexico and are part of a broader systemwide challenge relating to the implementation of Seguro Popular is a question that deserves further attention. Future research should use mixed-methods approaches to gather information on the lived experiences of Mexican youth as they interact with their local health clinics, as well as provide quantitative data with larger and representative samples to support qualitative findings. Such studies should incorporate culturally appropriate assessments of functional and critical HL so meaningful interventions aimed at reducing health disparities can be created. In brief, the positive changes taking place within the Mexican healthcare system should be accompanied by efforts to ensure at-risk populations—such as youth—are able to access, understand, and use the new resources at their disposal.

## Figures and Tables

**Table 1:** Descriptive statistics.

Variables	*N*	Mean	SD	Range
Gender (female = 48%)	112	0.52	0.5	0–1
Age	110	13.20	0.99	11–16
Ever taken a health calls (0 = no)	109	0.64	0.48	0–1
Year in school	91	8.15	0.93	7–10
Grade point average	110	3.22	0.67	0–4
Parent education level ^a^	104	0.73	1.49	0–6

^a^ 0 = less than high school; 1 = high school or equivalent; 2 = some college; 3 = associate’s degree; 4 = bachelor’s degree; 5 = master’s degree; 6 = doctorate or professional degree.

## Appendix A

- Where do you go for health information?
  ○ [For those who said “parents”] If you had a question about your health that your parents could not answer, where would you go for health information?
- Has it ever been difficult to obtain health information? If so, what types of challenges or barriers have you faced when seeking health information?
- In going to the doctor and speaking with them about your health, what challenges have you faced?
- In your opinion, when is it important to see a doctor or health professional?
- How easy or difficult is it to understand the health information you are given when you go to the doctor?
- How much do family members (e.g., mom, dad, siblings, aunt/uncle) assist you in setting up appointments, visiting with the doctor, and understanding the health instructions you are given?
- Have you ever been taught how to take care of your own health needs? If so, who taught you and what did you speak about?
- If you were to have a health problem that required medical attention would you know how to independently obtain the medical services you need without assistance from family or friends?
- What are your overall impressions of health services and healthcare in your community?
